# Lipid Rafts in Mast Cell Biology

**DOI:** 10.1155/2011/752906

**Published:** 2011-02-10

**Authors:** Adriana Maria Mariano Silveira e Souza, Vivian Marino Mazucato, Maria Célia Jamur, Constance Oliver

**Affiliations:** Departamento de Biologia Celular e Molecular e Biagentes Patogênicos, Faculdade de Medicina de Ribeirão Preto, Universidade de São Paulo, Avenida Bandeirantes, 3900, 14049-900 Ribeirão Preto, Brazil

## Abstract

Mast cells have long been recognized to have a direct and critical role in allergic and inflammatory reactions. In allergic diseases, these cells exert both local and systemic responses, including allergic rhinitis and anaphylaxis. Mast cell mediators are also related to many chronic inflammatory conditions. Besides the roles in pathological conditions, the biological functions of mast cells include roles in innate immunity, involvement in host defense mechanisms against parasites, immunomodulation of the immune system, tissue repair, and angiogenesis. Despite their growing significance in physiological and pathological conditions, much still remains to be learned about mast cell biology. This paper presents evidence that lipid rafts or raft components modulate many of the biological processes in mast cells, such as degranulation and endocytosis, play a role in mast cell development and recruitment, and contribute to the overall preservation of mast cell structure and organization.

## 1. Introduction

Mast cells, like blood cells, are derived from pluripotent bone marrow hematopoietic stem cells but, unlike blood cells, they leave the bone marrow as progenitors and migrate into virtually all vascularized tissues to complete their differentiation under the influence of factors present at each tissue site. It is the microenvironment surrounding the mast cells that determines their mature phenotype [1–6]. Mast cells are effector cells of allergic and anaphylactic reactions and play a role in many physiological and pathological processes [7, 8]. Recently, they have gained new importance as immunoregulatory cells with the recognition that they are a major source of cytokines and chemokines and play roles in both innate and adaptive immunities [7, 9, 10]. Although mast cells may be activated by a number of stimuli and pathways [11, 12], the major mechanism for their activation and subsequent degranulation is through the high-affinity receptor for immunoglobulin E (Fc*ε*RI), present in the plasma membrane of mast cells, epidermal Langerhans cells, eosinophils, and basophils [13]. Fc*ε*RI is expressed as a heterotetrameric structure composed of one *α* subunit with an extracellular domain that binds IgE, a four-transmembrane-spanning *β* subunit, and two identical disulphide linked *γ* subunits [14–17]. The *β* subunit serves as an important amplifier of IgE and antigen-induced signaling events. Furthermore, the *γ* subunits are essential for initiating signaling events downstream of Fc*ε*RI [17, 18]. The carboxyl terminal cytoplasmic domains of both the *β* and *γ* subunits contain an immunoreceptor tyrosine-based activation motif (ITAM), common to all multisubunit immune recognition receptors, that is critical for cell activation. Because the receptor subunits lack any known enzymatic activity, Fc*ε*RI must rely on associated molecules for transducing intracellular signals [16, 19, 20]. Mast cell activation is initiated by the binding of oligomeric antigens to receptor-bound IgE, which crosslinks Fc*ε*RI and results in its aggregation. The first recognized biochemical event of the cytoplasmic signal transduction cascade involves phosphorylation, presumably by Lyn, of two conserved tyrosine residues within the ITAMs of both *β* and *γ* subunits of the receptor. The tyrosine-phosphorylated ITAMs create a novel binding surface that is recognized by additional cytoplasmic signaling molecules, such as the protein tyrosine kinase Syk which binds mainly to the *γ* subunit, via its tandem Src homology 2 (SH2) domains. This interaction results in a conformational change in Syk, followed by its activation and autophosphorylation. This results in an increased kinase activity that rapidly shifts the equilibrium of the cell from a resting state (where phosphorylation and dephosphorylation activities are approximately equal) to an activated state (where phosphorylation activity increases exponentially and cannot be counteracted by dephosphorylation). This Syk-mediated signal amplification results in a direct or indirect activation of several proteins, including linker for activation of T cells (LAT), Vav, phospholipase C-*γ*1 (PLC-*γ*1), and PLC-*γ*2. Finally, downstream activation results in an increase in intracellular calcium levels, activation of other enzymes and adaptors, and rearrangement of the cytoskeleton that culminates in the release of three classes of mediators: (1) preformed mediators (stored in secretory granules), such as histamine, heparin, *β*-hexosaminidase, neutral proteases, acid hydrolases, major basic protein, carboxypeptidases, and some cytokines and growth factors, (2) newly formed lipid mediators, such as prostaglandins and leukotrienes, and (3) newly synthesized mediators, that include growth factors, cytokines, and chemokines [14, 21, 22]. Accumulating evidence suggests that lipid rafts or raft components play a pivotal role in signal transduction via Fc*ε*RI in mast cells and that the organization of various molecules in lipid rafts could modulate many biological processes in these cells. 

**Figure 1:**
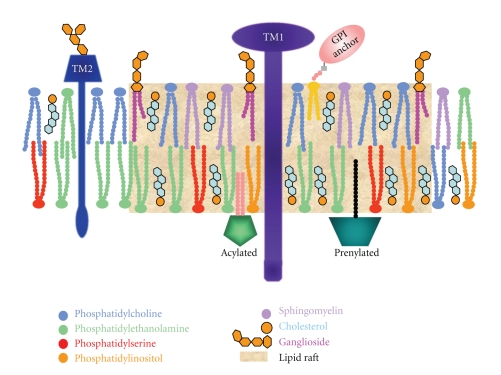
Diagrammatic representation of a lipid raft. Lipid rafts are enriched in cholesterol, sphingolipids, and gangliosides. GPI anchored proteins, sphingomyelin, phosphatidylcholine and gangliosides are present in the outer membrane leaflet. Prenylated proteins, acylated proteins, phosphatidlyserine, and phosphatidylethanolamine are present in the inner leaflet. Cholesterol is present in both leaflets and functions as a space filling molecule under the sphingolipid head groups. TM1, TM2, Transmembrane proteins 1 and 2.

Lipid rafts, present in all eukaryotic cells, are currently defined as dynamic-ordered nanoscale assemblies of proteins and lipids of the plasma membrane and other intracellular membranes, such as Golgi membranes, that associate and dissociate on a subsecond timescale [4, 23, 24]. They contain high levels of cholesterol, sphingolipids (such as sphingomyelin), and gangliosides. Lipid rafts selectively concentrate glycosylphosphatidylinositol- (GPI-) anchored proteins on their outer side and proteins anchored by saturated palmitoyl or myristoyl groups and cholesterol-binding proteins on the cytoplasmic side [25–29]. Their lipid composition (Figure 1), with a preponderance of longer saturated hydrocarbon chains that potentiate interdigitation between leaflets [30] and favors interaction with cholesterol [31], allows cholesterol to be tightly intercalated. Lipid rafts are highly organized and probably exist in a liquid-ordered (*l*_*o*_) phase, different from the rest of the plasma membrane which consists mainly of phospholipids (with unsaturated tails) in a liquid-disordered (*l*_*d*_) phase [32]. The extent of packing depends on the degree of saturation. The cis double bond present on unsaturated lipids introduces a rigid bend in the hydrocarbon tail which interferes with the tight packing and results in less stable aggregates [33]. Lipid rafts are characterized by high melting temperature and a resistance to solubilization in nonionic detergents such as Triton X-100, at low temperature [34]. They are dynamic in that both proteins and lipids can move in and out of raft domains with different partitioning kinetics [28], as well as by coalescing or by breaking up into smaller units [29]. Lipid rafts can also form stabile platforms that are important in signaling, viral infection, and membrane trafficking [24]. Despite a body of evidence supporting the existence of raft domains, the raft concept is still being debated [35] because the mechanisms that govern the associations among sphingolipid, cholesterol, and specific membrane proteins in live cell membranes remain unclear [36]. The controversy is largely due to the lack of standardized methodology for lipid raft studies and the difficulty in proving definitively that rafts exist in living cells without causing significant nonphysiological perturbations by using low temperatures or by extensive cross-linking [37]. The majority of the studies involving lipid rafts begin with detergent solubilization of whole cells followed by sucrose density gradient centrifugation and the recovery of detergent-resistant membranes from the light fractions of the gradient [19, 20]. However, the analysis of density gradient centrifugation experiments remains controversial because there is an indication that detergents may force associations between components that are not colocalized in intact cells [38]. Fractionation results are also known to be severely altered by varying the concentration of Triton X-100 [39, 40], by the use of different detergents, [41, 42], or by omission of detergents in general [43–45]. Another difficulty has been demonstrating the coexistence of *l*_*o*_ and *l*_*d*_ phases in live cells. However, technological advances have produced compelling data that self-organization of lipids and proteins can induce subcompartmentalization that organizes the bioactivity of cell membranes [31]. Recently, the lipid-based phase separation into liquid-ordered-like and liquid-disordered-like phases has been seen in giant plasma membrane vesicles (GPMVs) obtained by chemically induced blebbing from cultured cells [46, 47] or by using cell swelling to generate plasma membrane spheres (PMS) [48]. In 2010, Johnson et al. [49] using GPMVs showed that peripheral protein binding may be a regulator for lateral heterogeneity *in vivo. *These new approaches are very promising, allowing studies of the lipid domains in the absence of detergents and other perturbations of membrane structure. Advances in imaging and studies with improved integrated methodologies, such as flotation of detergent-resistant membranes, antibody patching and immunofluorescence microscopy, immunoelectron microscopy, chemical crosslinking, single fluorophore tracking microscopy, photonic force microscopy, spectrofluorimetry, mass spectrometry, and fluorescence resonance energy transfer (FRET) are now providing insights into the existence and behavior of lipid rafts [2, 16, 24, 50–56]. 

The lipid microdomains are variable in stability, size, shape, lifetime, and molecular composition [29, 37]. Due to differing molecular composition, studies of lipid rafts have also been complicated by imprecise nomenclature [24]. For example, caveolae was synonymous with lipid rafts for many years. In 1998, Harder et al. [57], using a cell system lacking caveolae, demonstrated that raft and nonraft markers segregated in the same cholesterol-dependent way in the absence of caveolae. These results showed that clustered raft markers segregate away from nonraft proteins in a cholesterol-dependent, but caveolin independent manner [56]. Today caveolae are considered a subset of lipid rafts [16, 58].

Membrane rafts in most cell types are enriched with signaling molecules by virtue of the affinity of signaling proteins including transmembrane receptors, GPI anchored proteins, G proteins, RhoA and Src kinases for rafts [1, 35]. The number of proteins reported to be regulated by specific lipid interaction is steadily increasing, but the precise structural mechanisms behind specific binding and receptor regulation in membranes remain uncharacterized [56]. A wealth of biochemical and genetic data have lent credence to the notion that raft function as a specialized signaling platform in cell membranes [59–65]. Most likely, the function of rafts is aided by stimulation-induced association and recruitment of various molecules with raft affinity, as well as varying degrees of raft engagement with the cytoskeleton [3, 4, 29]. Lipid rafts are also thought to be important sites for protein tyrosine kinase-mediated protein-protein interactions that are involved in the initiation of receptor signaling pathways [5, 6, 16]. It is well known that, in the case of tyrosine kinase receptors, adaptors, scaffolding proteins, and enzymes are recruited to the cytoplasmic side of the plasma membrane as a result of ligand binding to form a signaling complex [66]. If receptor activation takes place in an ordered lipid raft, the signaling complex is protected from other proteins, such as membrane phosphatases, localized in the disordered region of the plasma membrane, that otherwise could affect the signaling process [35, 51, 67]. Lipid rafts are implicated in the function of diverse signaling pathways such as those mediated by growth factors, morphogens, integrins [16] and antigen receptors on immune cells, including mast cells [68–71]. The structural basis for the association of Fc*ε*RI with lipid rafts is partially understood and appears to involve the transmembrane segments of Fc*ε*RI *α* and/or *γ* subunits. However, the structural features of Fc*ε*RI that mediate the detergent-sensitive interaction with lipid rafts occur selectively but not uniquely with this receptor [39]. Both *β* and *γ* subunits are palmitoylated, which could facilitate their association with lipid rafts [72].

Studies have shown that establishing and maintaining lipid rafts is important for many biological processes besides cell signaling [73, 74]. These membrane microdomains have been implicated in such processes as exocytosis, endocytosis, membrane trafficking, and cell adhesion. The structure-function relationship of lipid rafts or rafts constitutes are important in various aspects of mast cell biology.

## 2. Morphology

**Figure 2:**
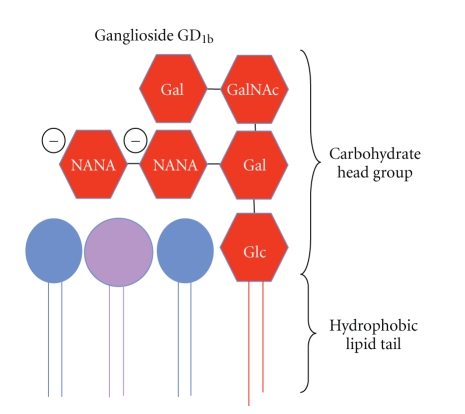
Diagrammatic representation of ganglioside GD_1b_. The ganglioside is composed of a carbohydrate head group and a hydrophobic lipid tail.

The ability to form lipid rafts appears to be important for maintaining the typical morphology of mast cells. Gangliosides (Figure 2), lipid raft components, are complex glycosphingolipids that are ubiquitous membrane constituents [5, 75–77] and seem to be structurally important for lipid raft assembly and function. The rigid structural nature of the ceramide anchor in gangliosides, coupled with the ability of sphingolipids to associate with cholesterol, is thought to drive the assembly of lipid rafts [16, 78]. 

**Figure 3:**
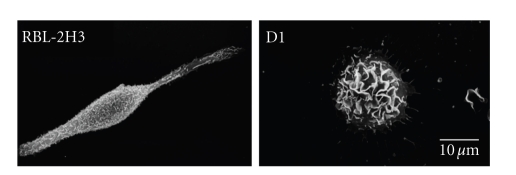
Ganglioside-deficient D1 cells have an altered morphology. By scanning electron microscopy, RBL-2H3 cells are spindle shaped and their surface is covered with short microvilli. In contrast, D1 cells are rounded and their surface is covered with large membrane ruffles.

The influence of gangliosides and/or lipid rafts on cell structure and organization was examined [79] using a ganglioside-deficient cell line, D1, and the parent cell line, RBL-2H3, a cell line with homology to mucosal mast cells [80–84]. The D1 cell line is deficient in GM_1_ gangliosides and in mast cell specific *α*-galactosyl derivatives of the ganglioside GD_1b_. The *α*-galactosyl derivatives of the ganglioside GD_1b_, antigens I and II, contain, respectively, one and two additional *α*-galactosyl residues when compared with GD_1b_. These unique gangliosides are present on the surface of rodent mast cells and are specifically recognized by the monoclonal antibody (mAb) AA4 [85]. These gangliosides derived from GD_1b_ have been identified as components of lipid rafts in the plasma membrane of RBL-2H3 cells [86, 87]. The mutant cell line D1 showed a cellular morphology which is distinct from RBL-2H3 cells (Figure 3), suggesting that the gangliosides are important in the maintenance of normal cell morphology. 

**Figure 4:**
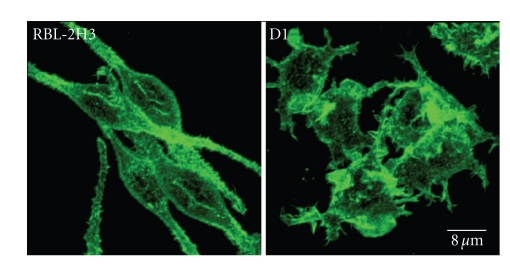
The F-actin distribution in RBL-2H3 and D1 cells reflects their morphology. Actin filaments in RBL-2H3 cells lie under the plasma membrane following the spindle shape of the cells and in association with microvilli. The actin cytoskeleton is altered in D1 cells and the actin filaments are concentrated in large membrane ruffles. The cells were fixed, permeabilized, and stained with phalloidin conjugated to Alexa 488. Samples were examined using a Leica TCS-NT laser scanning confocal microscope.

The morphological changes observed in D1 cells could be related to the lipid composition of these cells. This cell line presents a large decrease in glycosphingolipids, such as GM_1_ and the *α*-galactosyl derivatives of the ganglioside GD_1b_, which may affect many physicochemical properties of the plasma membrane. According to Kato et al. [88], the lipid composition could influence membrane stability, membrane fluidity, lipid packing, bilayer curvature, and hydration elasticity, as well as anchorage of the cytoskeleton to the plasma membrane. Silveira e Souza et al. [79] also observed that the D1 cells showed an abnormal distribution of actin filaments and microtubules. A growing body of evidence indicates that lipid rafts are essential for membrane-cytoskeleton coupling, and the association of Lyn and other raft markers with crosslinked Fc*ε*RI is regulated by interactions with F-actin [89–91]. It is possible that in the D1 mutant cells, the disorganization of both lipid rafts and actin filaments (Figure 4) leads to impaired degranulation after Fc*ε*RI stimulation [79, 87]. Furthermore, the actin cytoskeleton is known to participate in regulating and activating raft-associated signaling events [92–94].

The factors that govern the formation of lipid rafts continue to be elucidated, but lipid raft formation often requires actin filaments. The connection between lipid raft proteins and actin filaments can affect the lateral distribution and mobility of these membrane proteins [59, 95]. The extent to which the actin cytoskeleton participates in the formation of membrane rafts is not yet established. Han et al. [96] observed that perturbations in the actin filaments (with cytochalasin D and latrunculin A) affect the organization of lipid rafts in RBL-2H3 cells. Importantly, the actin cytoskeleton is a dynamic structure that changes in response to extracellular signals, and it may therefore represent one mechanism for governing the establishment and distribution of lipid rafts in the plasma membrane [97]. Chichili and Rodgers [98] showed that lipid rafts may be structured by a synergistic interaction between the cortical actin filaments and the lipid rafts themselves, and that many of the structural and functional properties of rafts require an intact actin cytoskeleton. An important regulator of membrane-cytoskeleton interactions is the phosphoinositide PIP2, which is a minor lipid component of the plasma membrane that is known to regulate the organization of the actin cytoskeleton and in particular the formation of actin-membrane linkages [99]. PIP2 also serves as a cofactor for many of the proteins that anchor actin filaments to the plasma membrane [99, 100]. Protein binding to PIP2 often occurs through a PIP2-specific recognition sequence, in many cases represented by a PIP2-specific pleckstrin homology (PH) domain [101–103]. Some actin binding proteins (ABPs) are thought to link actin filaments and PIP2-enriched rafts. Gelsolin is one of the ABPs present in lipid rafts [104]. Microtubules are one of the major determinants of cell shape and polarity [105, 106]. In the ganglioside-deficient D1 cells, the arrangement of microtubules was completely disorganized. The results from this study have demonstrated that the abnormal morphology observed in the mutant cell line could be related to the decrease in gangliosides that leads to lipid raft disorganization [79].

## 3. Endocytosis

**Figure 5:**
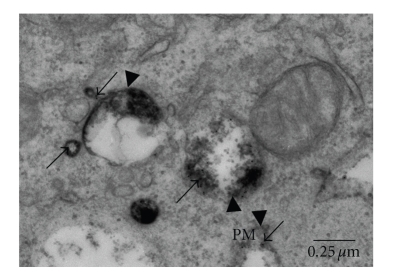
The gangliosides follow the same endocytic pathway as Fc*ε*RI. At 15 minutes of incubation with both mAb BC4-gold (which recognizes the *α* subunit of Fc*ε*RI) and mAb AA4-HRP (which recognizes gangliosides derived from GD_1b_), the BC4-gold (arrows) and AA4-HRP (arrowheads) are colocalized in early endosomes adjacent to the plasma membrane (PM).

When the concept of lipid rafts and the mobility of proteins in the plasma membrane originated, it was observed that plasma membrane associated proteins could suffer a selective reorganization followed by internalization of these proteins [107–109]. Receptor-mediated endocytosis, including endocytosis of Fc*ε*RI, is a temporally and spatially organized process [22, 110]. After activation, crosslinked Fc*ε*RI is endocytosed through clathrin-coated vesicles and transported by the endosomal system for eventual degradation in lysosomes [111–113]. In unstimulated mast cells, Fc*ε*RI is dispersed throughout the plasma membrane but upon activation the receptors rapidly aggregate and can be found on the cell surface in lipid rafts in association with GM_1_ [114, 115], gangliosides derived from GD_1b_, protein tyrosine kinase Lyn and LAT, [22, 39, 86]. However, only when the mast cells are activated via Fc*ε*RI does a significant internalization of the GD_1b_ derivatives occur [22, 116]. The endocytosis process itself may play an important role in signal transduction [110, 117]. Oliver et al. [22] showed that upon activation of Fc*ε*RI, the gangliosides derived from GD_1b_ are internalized together with the receptor, following the same pathway to lysosomes (Figure 5). This may facilitate the structural preservation of signaling complexes and the prolongation of the signal since these gangliosides and the Fc*ε*RI are associated in lipid rafts. 

In view of the importance of lipid raft integrity for efficient receptor endocytosis, it has been observed that the Fc*ε*RI ubiquitination is a key mechanism for the regulation and control of antigen-dependent endocytosis of receptor complexes [118]. Moreover, it has been demonstrated that ubiquitin ligases Cbl and Nedd4 are recruited into lipid rafts upon IgE triggered cell signaling [119]. Nedd4 was shown to ubiquitinate membrane receptors [120]. The ubiquitin Cbl is a good candidate to mediate Fc*ε*RI ubiquitination since it participates in various functions such as cis-and-trans-ubiquitination [121]. It is phosphorylated upon Fc*ε*RI engagement [122] and negatively regulates Syk kinase [123]. Molfetta et al. [124, 125] suggested that the recruitment of engaged Fc*ε*RI subunits into lipid rafts precedes their ubiquitination, and that integrity of lipid rafts is required for receptor ubiquitination and endocytosis, contributing to the down-regulation of Fc*ε*RI-mediated signaling.

## 4. Signal Transduction

In mast cells, the first signaling complex convincingly shown to involve lipid rafts was immunoglobulin E (IgE). IgE signaling was initially thought to be based on protein-protein interactions alone, but several observations indicated that lipid rafts are involved in this process [37, 68, 126–131]. The first hint came from the finding that Fc*ε*RI is soluble in Triton X-100 at steady state but becomes insoluble in low concentrations of this detergent after crosslinking [68]. Moreover, in unstimulated cells, Fc*ε*RI is dispersed throughout the plasma membrane, but upon activation rapidly aggregates [115, 132] and can then be found on the cell surface in association with the ganglioside GM_1_ [57, 114, 133] and GPI-anchored proteins [89, 134]. Despite numerous studies on mast cell activation through Fc*ε*RI, the detailed mechanism by which cross-linking promotes the initial phosphorylation by Lyn and the molecular mechanisms for Lyn activation are still unclear [67, 77, 135, 136]. Davey et al. [37] suggested that protein-protein interaction (IgE-Fc*ε*RI cross-linking) recruits essential signaling proteins and lipid molecules into more ordered domains that serve as a platform for signaling.

An approach intensively used to better understand the role of lipid rafts in Fc*ε*RI-mediated signaling has been the study and/or the manipulation of the lipid constituents of rafts, such as cholesterol and gangliosides [16, 87, 136]. Methyl-*β*-cyclodextrin (M*β*CD), a carbohydrate molecule with a pocket for binding cholesterol, [16] is extensively used to deplete the surface cholesterol and subsequently disrupt lipid rafts. M*β*CD has been used to study the role of lipid rafts in Fc*ε*RI-mediated signaling, particularly in early events of signal transduction such as tyrosine phosphorylation of Fc*ε*RI by Lyn [136]. Sheets et al. [86] have demonstrated that phosphorylation of Fc*ε*RI proceeds in a cholesterol-dependent manner and that cholesterol depletion reduces stimulated tyrosine phosphorylation of Fc*ε*RI. In parallel to its inhibition of tyrosine phosphorylation, cholesterol depletion disrupts the interactions of aggregated Fc*ε*RI and Lyn in intact cells. Cholesterol repletion restores receptor phosphorylation together with the structural interactions, providing strong evidence that lipid raft structure, maintained by cholesterol, plays a critical role in the initiation of Fc*ε*RI signaling. Cholesterol depletion by M*β*CD in RBL-2H3 cells also reduced the release of *β*-hexosaminidase activity in cells stimulated via Fc*ε*RI [87, 88, 137, 138]. These data suggest that the cholesterol depletion by M*β*CD affects the IgE signaling due to the disruption of lipid rafts and consequently results in a failure to form a signaling complex. Moreover, Young et al. [67] showed evidence that Lyn isolated in lipid rafts has substantially higher Lyn kinase activity than Lyn outside of these membrane microdomains. These data suggest that some unknown components in lipid rafts may influence the kinase activity of Lyn [136] and subsequently Fc*ε*RI signal transduction.

Flotillin-1 is another constituent of lipid rafts [139, 140]. It was initially identified as a caveolae-associated membrane protein and is a marker protein of lipid rafts, but its physiological role is still not clear. Kato et al. [136] using flotillin-1 knockdown RBL-2H3 cells showed that flotillin-1 regulates the kinase activity of Lyn in mast cells. In the flotillin-1 knockdown cells, there was a significant decrease in Ca^2+^ mobilization, the phosphorylation of ERKs, tyrosine phosphorylation of the *γ*-subunit of Fc*ε*RI, and IgE-mediated degranulation. This study also showed that flotillin-1 is constitutively associated with Lyn in lipid rafts in RBL-2H3 cells, and that antigen stimulation induced an increase in flotillin-1 binding to Lyn, resulting in enhancement of the kinase activity of Lyn. These data suggest that this raft protein is an important component of Fc*ε*RI-mediated mast cell activation and regulates the kinase activity of Lyn in lipid rafts. 

The *α*-galactosyl derivatives of the gangliosides GD_1b_ also seem to be intimately involved with signaling through Fc*ε*RI. Although the functional role of these gangliosides is not clear, previous studies have shown that when the *α*-galactosyl derivatives of ganglioside GD_1b_ are bound by mAb AA4, histamine release was inhibited in a time- and concentration-dependent manner. Binding of mAb AA4 to RBL-2H3 cells resulted in an increase in intracellular calcium, phosphatidylinositol hydrolysis, and a redistribution of PKC. However, the magnitude of these changes was less than those after Fc*ε*RI aggregation, and unlike Fc*ε*RI activation, these changes were not accompanied by histamine release [81]. The derivatives of the ganglioside GD_1b_ coprecipitated with the Src family tyrosine kinase Lyn and that in spite of the fact that mAb AA4 binds to sites close to Fc*ε*RI the association between Lyn and these gangliosides was not mediated by Fc*ε*RI. The association of Lyn with these gangliosides is much stronger than the association of Lyn with Fc*ε*RI. These associations suggest that a complex of molecules that includes gangliosides, Fc*ε*RI, and Lyn is essential for modulation of signal transduction in mast cells [81, 141–144].

Furthermore, analysis of the subcellular distribution of the gangliosides recognized by mAb AA4 and of Fc*ε*RI on sucrose gradients showed that, following Fc*ε*RI activation, there was a shift in the distribution of the gangliosides to the lipid raft fractions [22, 87]. The movement of these gangliosides into the lipid rafts may be another mechanism that regulates signal transduction in mast cells.

 As previously stated, using a cell line deficient in the *α*-galactosyl derivatives of ganglioside GD_1b_, as well as the parent cell line, RBL-2H3, Silveira e Souza et al. [87] demonstrated and confirmed the importance of these gangliosides for lipid raft organization and consequently for Fc*ε*RI-mediated degranulation in rodent mast cells. In this study, the authors observed a decreased release of *β*-hexosaminidase activity in the mutant cell line after Fc*ε*RI stimulation, but not after exposure to calcium ionophore. These results show that release of *β*-hexosaminidase activity is calcium-dependent and furthermore indicated that the mutant cell line possesses the capacity to degranulate. Moreover, reduced release of *β*-hexosaminidase activity in RBL-2H3 cells treated with compounds that inhibit ganglioside synthesis was also observed.

In addition to lipid raft assembly, another possible role for the mast cell-specific gangliosides in signal transduction could be to facilitate the association of Lyn with Fc*ε*RI. Because Fc*ε*RI itself has no intrinsic kinase activity, the tyrosine phosphorylations induced by receptor cross-linking could be a secondary event that occurs after aggregation of Fc*ε*RI and its movement into lipid rafts [143]. Therefore, these lipid raft complexes that include gangliosides, associated proteins, such as Lyn, LAT, flotillin-1 and Fc*ε*RI, have an important role in receptor-mediated signal transduction. 

Recently, Fifadara et al. [8] reported that mast cells produce structures such as cytonemes or tunneling nanotubes used for intercellular communication and that intercellular communication may be important during allergic and inflammatory responses following costimulation of Fc*ε*RI and CCR1. Albeit the process of cytoneme formation remains poorly understood, the fact that cholesterol depletion reduced the formation of cytonemes suggests that lipid rafts may participate in cytoneme formation in mast cells, either by promoting membrane integrity or by participating in cell signaling.

## 5. Mast Cell Development and Recruitment

The expression of the *α*-galactosyl derivatives of the ganglioside GD_1b_ on the mast cell surface also appears to be related to mast cell development and recruitment. Previous studies using mAb AA4 showed that the *α*-galactosyl derivatives of the ganglioside GD_1b_ were present only in mast cells and not in any other cell type in all 23 rat tissues examined [81, 85]. However, in bone marrow, a population of large, poorly differentiated cells, presumably immature mast cells were also stained with mAb AA4 [81]. Later these cells were indeed shown to be very immature and immature mast cells [145, 146]. Since the heterogeneity of the maturing mast cells makes them impossible to separate from other cells on the basis of their density and mAb AA4 binds only to cells which can be identified as mast cells [146, 147], the gangliosides recognized by mAb AA4 may be considered a powerful marker for rodent mast cells.

The ability to characterize the maturation of bone marrow-derived and peritoneal mast cells has been impaired both by the lack of mast cell-specific markers and by the inability to rapidly and efficiently separate mast cells in all stages of maturation from a mixed population of cells [148]. Using mAb AA4 conjugated to tosylactivated Dynabeads 450, Jamur et al. [145] successfully separated mast cells from rat bone marrow and the peritoneal lavage. They [146] then went on to isolate and characterize bone marrow mast cells at various stages of maturation. In this study, the very immature mast cells, which had not been previously described, were identified by the presence of the derivatives of the ganglioside GD_1b_ on their surface. These cells which could not be recognized as mast cells by standard cytological methods contained only a few small cytoplasmic granules. On the other hand, undifferentiated mast cell precursors in the bone marrow do not express the *α*-galactosyl derivatives of the ganglioside GD_1b_ recognized by mAb AA4. These gangliosides begin to be expressed on the cell surface jointly with Fc*ε*RI and at the same time as the initiation of the formation of cytoplasmic granules in very immature mast cells. The gangliosides derived from GD_1b_ continue to be expressed by mast cells in all stages of maturation [149]. These data suggest that mast cell lipid rafts or raft constitutes are related to mast cell maturation and function.

## 6. Conclusions

Several aspects of raft structure and function in mast cell biology still need to be elucidated. Undoubtedly, lipid rafts and their constitutes play a role in many aspects of mast cell biology, such as activation through Fc*ε*RI, morphology, endocytosis, and maturation. Further research to better define the role of lipid rafts in mast cells could offer novel targets for immunotherapies and treatment of diseases in which mast cells and/or their mediators are involved.
